# Trigeminal Meningioma in a Patient with Tardive Dyskinesia as Only Symptom

**DOI:** 10.1155/2018/6175165

**Published:** 2018-12-31

**Authors:** Maria-Gabriela Catană, Andreea-Alina Dan, Corina Roman-Filip

**Affiliations:** ^1^Clinical County Emergency Hospital of Sibiu, Department of Neurology, Romania; ^2^Clinical County Emergency Hospital of Sibiu, Department of Radiology, Romania; ^3^Lucian Blaga University of Sibiu, Faculty of Medicine, Romania

## Abstract

Most meningiomas are benign, encapsulated tumors (95% of the cases), generally undergoing a limited number of genetic aberrations. We present the case of a 74-year-old patient with no significant pathological history, who is admitted to the neurology ward for orofacial dyskinesias accompanied by hypoesthesia in the left hemiface, a symptomatology that had started insidiously about two months before and worsened progressively over the past 3 weeks. A cerebral MRI was performed which revealed a small mass with discrete T2 hyperintensity and T1 iso-signal compared to the gray matter located in the left pontine cistern, with a large, well-defined base at the level of the cerebral tentorium. The diagnosis of trigeminal meningioma was established and the treatment was started, after hearing the opinion of a neurosurgeon.

## 1. Introduction

Most meningiomas are benign, encapsulated tumors (95% of the cases), generally undergoing a limited number of genetic aberrations. Although they are the most common central nervous system tumors (20% of all central nervous system tumors), meningiomas have a rather poorly defined incidence, epidemiology, and prognosis [1]. Tumor localization is a critical factor in determining the prognosis, therapeutic options, and especially the degree of resectability of the tumor [2]. Despite benign tumors, meningiomas have been reported to shorten survival rates. The treatment of choice for meningioma is surgical resection, but when the tumor is difficult to approach anatomically, a symptomatic, conservative treatment is preferred.

## 2. Case Presentation

**Figure 1:**
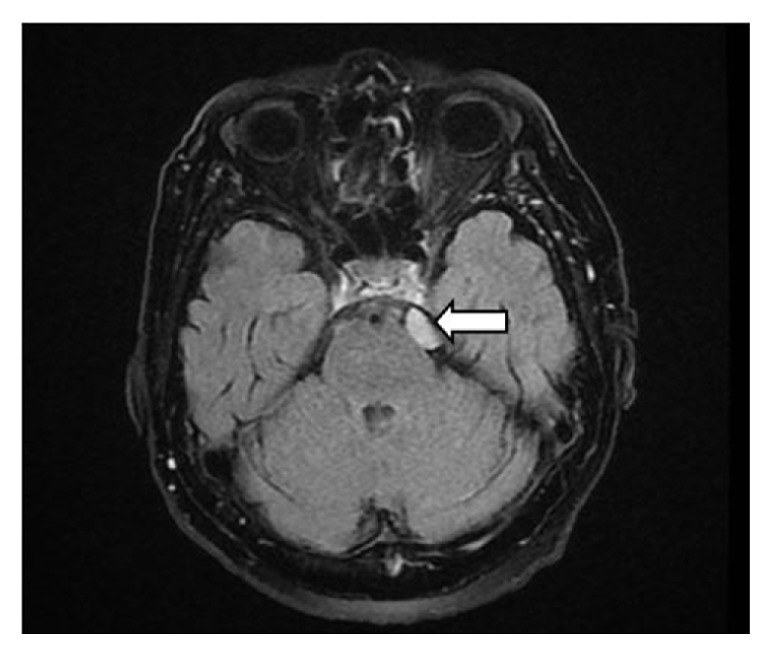
Trigeminal meningioma: cerebral MRI-T2 flair sequence.

**Figure 2:**
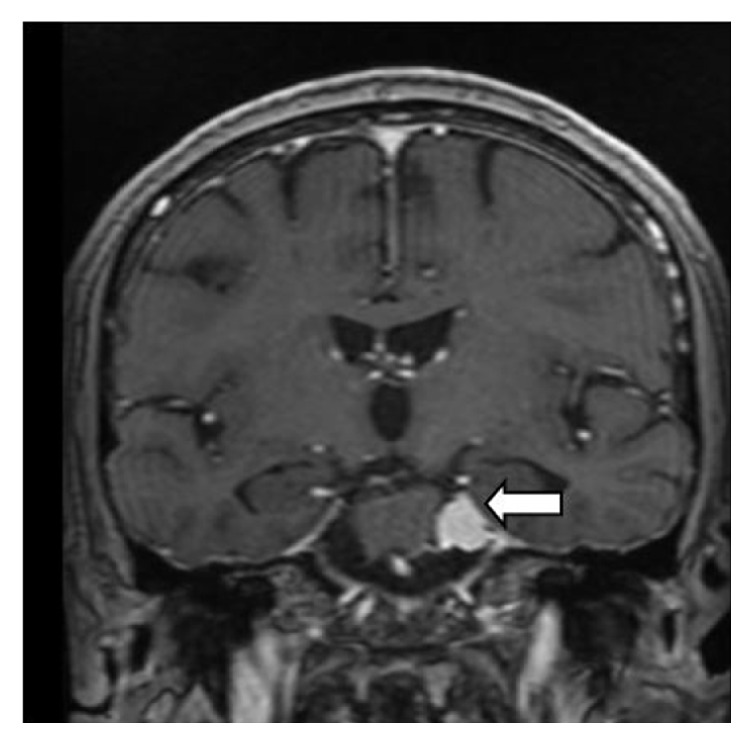
Cerebral MRI showing trigeminal meningioma: T1 coronal and sagittal sequence.

**Figure 3:**
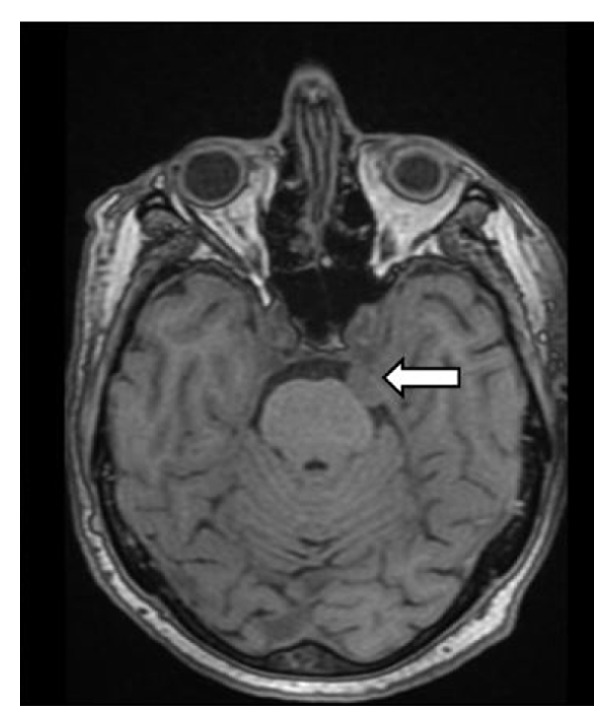
Cerebral MRI showing trigeminal meningioma: T1 coronal and sagittal sequence.

We present the case of a 74-year-old patient with no significant pathological history, who is admitted to the neurology ward for orofacial dyskinesias accompanied by hypoesthesia in the left hemiface, a symptomatology that had started insidiously about two months before and worsened progressively over the past 3 weeks. The objective general examination revealed a normal weight patient with a relatively good general condition when admitted to hospital, hemodynamically stable-blood pressure (BP) = 130/70 mmHg, AV = 72 bpm, and afebrile. The neurological examination revealed such changes as left hemifacial hypoaesthesia, orofacial dyskinesia, extrapyramidal syndrome, bradykinesia and bilateral hypokinesia without motor deficits or coordination disorders, without speech impediments, left plantar extension, and right plantar flexion. All laboratory test results were physiologically within limits, which helped exclude a number of causes of dyskinesia, such as thyroid pathology. However, the vast differential diagnosis of orofacial dyskinesia, which excluded use of neuroleptics, demonstrated that it was imperative to go to the next stage, namely, paraclinical tests. The EEG revealed no changes, so a cerebral MRI with contrast was performed which revealed a small mass with discrete T2 hyperintensity (Figure 1) and T1 iso-signal compared to the gray matter located in the left pontine cistern, with a large, well-defined base at the level of the cerebral tentorium, approximately 1.5/1/1.5 cm (diam AP/LL/CC) (Figures 2 and 3), homogeneous, marked as a gadolinophil tumor, with a visible “dural tail”, noninvasive, nondiffuse, with a slight mass effect on the pons, which indicate the presence of a meningioma. Mention should be made of the fact that the mass is directly connected to the cisternal portion of the trigeminal nerve, on its entrance to Meckel's cave. There were many well-defined, bilateral, periventricular, 6 mm, FLAIR, and T2 hyperintensity images, with no restriction in DWI/ADC, small microangiopathic ischemic demyelinated lesions.

The head MRI helped identify the cause of the extrapyramidal syndrome: periventricular ischemic lesions and the diagnosis established was vascular parkinsonian syndrome. The patient was referred to a neurosurgeon for a consult, with a view to making a therapeutic decision. His opinion was that, given the difficult approach to the meningioma, the best decision would be to attempt delaying the surgery. As a result, a symptomatic treatment was prescribed, which included 100 mg tiapride, 2 tablets a day. However, the patient did not respond favourably and the dyskinesia persisted. It was decided to discontinue the tiapride treatment and the patient was prescribed tetrabenazine 25 mg, 2 tablets a day (valbenazine is not available in Romania at the moment), to which he responded favourably. When the patient was discharged, the dyskinesia was much diminished and even absent at times, so the patient was advised to continue the tetrabenazine treatment, to repeat the brain MRI scan within 6 months, and then to see the neurosurgeon for reassessment. The images included in this article are from the MRI performed at 6 months after the diagnosis. We wanted to point out that the meningioma's dimensions remained the same, so we decided to continue with the medication and a next appointment was established in 6 months.

## 3. Discussion and Conclusions

Tardive dyskinesia occurs mainly in patients diagnosed with psychiatric pathologies such as schizophrenia or bipolar disorder and in patients who use neuroleptic medication.

Meningiomas are the most common type of brain tumors, with a prevalence of about 8-10 cases in 100,000 patients. Despite benign tumors, meningiomas have been reported to shorten survival rates. For example, a study was conducted in Finland on a cohort of 1986 patients, with a 31 year follow-up, and a survival rate of 86% was noted every 10 years. In the US, a comparative study was conducted which included 581 patients diagnosed with meningioma, with an 8-year survival rate of about 82% [1, 3], which was much lower than that of a cohort of subjects of the same gender and similar age, but with no tumor history.

Trigeminal meningioma is a tumor whose main symptom is trigeminal neuralgia, which, in 15% of the cases, is caused by the presence of a brain tumor [4, 5]. The typical symptoms of meningioma include epileptic seizures, headaches, nausea, vomiting, and behavioral or cognitive changes. None of the symptoms listed above were present in our patient. Despite being atypical, the only symptom attributed to the trigeminal meningioma in the present case was orofacial dyskinesia.

The parkinsonian syndrome, which, along with dyskinesia, initially appeared to be one of the symptoms of meningioma, was found to be caused by the presence of periventricular ischemic spaces. It is commonly known that dyskinesia is part of a neurological pathology that does not easily respond to treatment, but it almost completely disappeared in our patient following administration of tetrabenazine, 50 mg BID a day. Given the location of the tumor and the major risk of relapse, as demonstrated in recent studies (63% within 5 years after partial resection) [5], it was decided to delay surgery as long as the patient responded favourably to treatment.

The diagnosis was supported by the fact that the tumor was found to be compressing the bridge. Most likely there was predominant compression on the motor fibers of the trigeminal nerve. Our intention in presenting this case was to point out the fact that patients' symptoms can often be confusing. We often have to choose between several symptoms brought about by different causes.

We also need to keep in mind the fact that different pathologies can generate unexpected symptoms and we should take into account hypotheses that may sometimes seem impossible.

All in all, the present case report focuses on the fact that the eyes cannot see what the mind does not know. As we cannot establish a relation between pathology in the brain or brainstem and a clinical picture in a patient, imaging technique preferably an MRI scan with a focus on the course of the trigeminal nerve can be of utmost importance for the diagnosis of such cases. Follow-up scans are needed because meningioma can recur years or even decades after treatment.
